# Secondary distribution of HIV self-test kits by HIV index and antenatal care clients: implementation and costing results from the STAR Initiative in South Africa

**DOI:** 10.1186/s12879-023-08324-7

**Published:** 2023-06-01

**Authors:** Vincent Zishiri, Donaldson F. Conserve, Zelalem T. Haile, Elizabeth Corbett, Karin Hatzold, Gesine Meyer-Rath, Katleho Matsimela, Linda Sande, Marc d’Elbee, Fern Terris-Prestholt, Cheryl C. Johnson, Thato Chidarikire, Francois Venter, Mohammed Majam

**Affiliations:** 1 Ezintsha, a Sub-Division of Wits Reproductive Health and HIV Institute (Wits RHI), University of the Witwatersrand, Johannesburg, South Africa; 2 Department of Prevention and Community Health, Milken Institute of Public Health, George Washington University, District of Columbia, USA; 3 Department of Social Medicine, Ohio University Heritage College of Osteopathic Medicine, Dublin, USA; 4 London School of Hygiene and Tropical Medicine, London, UK; 5 Population Services International, Washington, USA; 6 Health Economics and Epidemiology Research Office (HE2RO), Department of Internal Medicine, Faculty of Health Sciences, University of the Witwatersrand, Johannesburg, South Africa; 7 Centre for Global Health and Development, Boston University, Boston, USA; 8 Global HIV, Hepatitis and STI Programmes, World Health Organization, Geneva, Switzerland; 9 National Department of Health, Johannesburg, South Africa

**Keywords:** HIV self-testing, Secondary distribution, HIV index clients, Men, Linkage to care, South Africa

## Abstract

**Background:**

Partner-delivered HIV self-testing kits has previously been highlighted as a safe, acceptable and effective approach to reach men. However, less is known about its real-world implementation in reaching partners of people living with HIV. We evaluated programmatic implementation of partner-delivered self-testing through antenatal care (ANC) attendees and people newly diagnosed with HIV by assessing use, positivity, linkage and cost per kit distributed.

**Methods:**

Between April 2018 and December 2019, antenatal care (ANC) clinic attendees and people or those newly diagnosed with HIV clients across twelve clinics in three cities in South Africa were given HIVST kits (OraQuick Rapid HIV-1/2 Antibody Test, OraSure Technologies) to distribute to their sexual partners. A follow-up telephonic survey was administered to all prior consenting clients who were successfully reached by telephone to assess primary outcomes. Incremental economic costs of the implementation were estimated from the provider’s perspective.

**Results:**

Fourteen thousand four hundred seventy-three HIVST kits were distributed – 10,319 (71%) to ANC clients for their male partner and 29% to people newly diagnosed with HIV for their partners. Of the 4,235 ANC clients successfully followed-up, 82.1% (3,475) reportedly offered HIVST kits to their male partner with 98.1% (3,409) accepting and 97.6% (3,328) using the kit. Among ANC partners self-testing, 159 (4.8%) reported reactive HIVST results, of which 127 (79.9%) received further testing; 116 (91.3%) were diagnosed with HIV and 114 (98.3%) initiated antiretroviral therapy (ART). Of the 1,649 people newly diagnosed with HIV successfully followed-up; 1,312 (79.6%) reportedly offered HIVST kits to their partners with 95.8% (1,257) of the partners accepting and 95.9% (1,206) reported that their partners used the kit. Among these index partners, 297 (24.6%) reported reactive HIVST results of which 261 (87.9%) received further testing; 260 (99.6%) were diagnosed with HIV and 258 (99.2%) initiated ART. The average cost per HIVST distributed in the three cities was US$7.90, US$11.98, and US$14.81, respectively.

**Conclusions:**

Partner-delivered HIVST in real world implementation was able to affordably reach many male partners of ANC attendees and index partners of people newly diagnosed with HIV in South Africa. Given recent COVID-19 related restrictions, partner-delivered HIVST provides an important strategy to maintain essential testing services.

**Supplementary Information:**

The online version contains supplementary material available at 10.1186/s12879-023-08324-7.

## Introduction

National patterns of HIV testing services (HTS) uptake in sub-Saharan Africa (SSA), including in South Africa, indicate that men have a lower HIV testing rate than women [1–4]. As a consequence, a high proportion of men living with HIV (MLWH) are unaware of their status and may engage in risky sexual behaviors that lead to HIV transmission [1]. The low rate of HTS uptake among men compared to women also results in higher HIV-related mortality among men due to late-stage diagnosis and initiating antiretroviral therapy (ART) with lower CD4 cell counts [5–7]. One promising strategy that has been shown to be effective in increasing HIV testing and feasible in reaching men is HIV self-testing (HIVST), which allows people to test for HIV in private versus at a healthcare facility or in the presence of a provider [8–13]. Men prefer HIVST over traditional testing approaches due to the lack of waiting time, confidentiality of results, and the autonomy HIVST provides reducing queues for facility-based HIV testing [14, 15]. HIVST implementation has been recommended by the World Health Organization (WHO) based on robust evidence from around the globe that highlighted a range of feasible and effective service delivery approaches – including partner-delivered HIVST or secondary distribution [11, 16].

Secondary HIVST distribution has mostly been used to reach men by providing women multiple HIVST kits to deliver to their male partners [8, 10]. For example, men in Zambia who were absent during household visits by a community HIV care provider were able to test at a different time by using HIVST kits that their partner received during the visit to deliver to their partners and other household members [9]. In a randomized controlled trial conducted in Malawi, men who received HIVST kits delivered by their female partners and a financial incentive or phone call reminder were more likely to test for HIV and seek follow-up services than men who received only a letter invitation to attend the clinic [10]. To date, most of the evidence for the feasibility and efficacy of secondary HIVST distribution is from randomized controlled trials and studies targeting male partners of women attending antenatal clinics (ANC) who are HIV-negative [8, 10, 17]. Only a couple of secondary HIVST distribution studies conducted in Malawi have provided HIVST kits to women newly diagnosed or living with HIV (i.e. index clients) to deliver to their male partners [18]. Female index clients have reported risk of violence and abandonment regarding secondary HIVST distribution to their partners, especially if the partner receives a positive self-test result [19]. Therefore, more implementation science research is needed to provide additional evidence on how to integrate index client-based secondary HIVST distribution safely in healthcare facilities and communities across different settings.

As part of the Self-Testing AfRica (STAR) Initiative in South Africa (SA) [20], we evaluated programmatic implementation of partner-delivered self-testing through antenatal care (ANC) attendees and index clients by assessing use, positivity, linkage and cost per kit distributed. The STAR Initiative in South Africa is part of the larger STAR project being implemented in multiple countries with the objectives of accelerating access to HIVST in low and middle income countries by creating an enabling environment with regard to HIVST policies, generating diverse demand through multiple distribution channels adapted to the needs of priority populations [21]. In South Africa, HIVST was included as a supplementary strategy in the National HIV Testing Services Policy in 2016 [22] and guidelines for HIVST were included in the South African National Strategic Plan for HIV, sexually transmitted infections and tuberculosis 2017–2022 [23]. Based on these guidelines, the SA STAR Initiative team implemented and evaluated different HIVST distribution strategies varying from community-based models (e.g., transport hubs, mobile, door-to-door), public sector models (e.g., pharmacy and workplace), and secondary distribution models with ANC and index clients to support the integration of HIVST in healthcare settings and other distribution sites [20]. In this manuscript, we report on the HIVST use by sexual partners of ANC and index clients, positivity, linkage and cost per kit outcomes for the secondary distribution model.

## Methods

### Settings

Secondary distribution of HIVST through community health clinic attendees was done in five clinics in the City of Johannesburg (CoJ) district and four clinics in City of Tshwane (CoT) both in the Gauteng Province as well as three clinics in Dr Kenneth Kaunda (DKK) district in North West Province of South Africa. CoJ, CoT, and DKK are three of the twenty-seven priority districts that account for 82% of the HIV burden in South Africa. Trained lay counsellors from the National Department of Health working at the clinics received standard training based on the HIVST curriculum developed in collaboration with the STAR consortium partners in South Africa.

### HIVST demand creation

Between April 2018 and December 2019, all clinic attendees were informed of the availability of HIVST kits that they could take home for their partners to use. Clinic attendees who were not ANC clients nor people living with HIV were informed of community outreach modalities, where they could receive HIVST. HIVST demand creation talks focused on the ease, convenience and confidentiality of conducting an HIVST and the need for male sexual partners of pregnant women and partners of people living with HIV to know their status and have the ability to access HIV prevention and treatment services. Information, education and communication materials on HIVST and answering questions about oral-HIVST (OraQuick Rapid HIV-1/2 Antibody Test, OraSure Technologies) were distributed to clinic attendees. Prior to provision of HIVST kits for partner’s use, HIVST kit use was demonstrated, and clinic attendees were provided with pre-packed kits for their partners in non-transparent bags. Emphasis was placed on clinic attendees to encourage their partners to read the user information sheet included with the HIVST kit to get more accurate performance of the test and interpretation of the results by the partner. Clinic attendees were encouraged to use the HIVST website link to view a demonstration video on testing at home.

### HIVST distribution

#### ANC clinic attendees

Up to three HIVST kits were offered for partners of women attending their ANC visit as part of post-test counselling in routine HTS irrespective of their HIV status. Women attending ANC visits were given brief messages on the benefits of partner testing during pregnancy and on how to perform the test and offer an HIVST kit to their male partner. Additional information on linkage to prevention and treatment services was also provided. HIVST kits were not offered to ANC clients if the partner had been diagnosed with HIV or if the partner had recently tested negative for HIV. Trained lay counsellors screened for clients’ risk of social harm upon kit delivery. Kits were not provided to clinic attendees who indicated that the offer of HIVST may not be well received by their partner, however information, education and communication materials on HIV prevention and testing, including HIVST; which could be shared with the partners were provided for clinic attendees.

### HIV index clients (people living with HIV)

HIVST kits were provided to all clients newly diagnosed with HIV or clients attending adherence clubs to take home and offer to their sexual partners who were unaware of their status. HIV index clients were exclusive to the ANC clinic attendees. Adherence clubs allow people living with HIV to collect their ARVs outside of their local clinics. Instead, they join an adherence club where they can collect their medication and join discussions about the issues of treatment adherence, adherence clubs were hosted at the clinic or at community settings were people living with HIV routinely collected their ARVs HIVST were not provided to index clients if they reported no current sexual partner or if they indicated a high risk of violence/harm.

### Outcomes

Primary outcomes were defined as (i) number of test kits distributed through secondary distribution, (ii) number of primary recipients successfully followed up, (iii) number of partners that accepted to use kits, (iv) number of partners that used HIVST kits. The denominator used for this measure was total number of test kits distributed for secondary distribution. Other outcomes included proportion of successfully reached self-reporting clinic attendees reporting use of HIVST by their sexual partners in both the ANC and HIV index testing clients; positivity rate and proportions of clinic attendees reporting their partners as having taken a confirmatory test on their reactive HIVST result and those subsequently initiating on ART.

### Data collection

Counsellors completed paper-based data collection forms (Additional file 1) which included client information and partner demographics. Clinic attendees who agreed to a telephonic follow-up call also provided their contact numbers. Trained linkage officers administered a standardized questionnaire to all consenting clinic attendees to assess HIVST use by their partners. The follow-up calls also sought to ascertain the partner's HIVST result and in the case of those who received a positive HIVST result, whether they received confirmatory testing and initiated ART. Up to 3 telephonic follow-up attempts were made at 2-, 4- and 6-weeks post kit distribution to consenting clinic attendees.

### HIVST distribution data analysis

Data analysis was conducted using STATA version 14.0 (Stata Corp LP, college station, TX)) and SAS 9.4 (SAS Institute, Cary NC). Population demographic characteristics of the partners of clinic attendees were summarized and using frequencies, proportions, median and interquartile range as appropriate frequencies and proportions of successful follow-up call rates amongst consenting clinic attendees were reported. Additionally, we analyzed in STATA the proportions of clinic attendees delivering kits to their sexual partners, proportions of partners using HIVST, and proportions of HIVST screened-positive clients attending clinics for confirmatory testing and subsequent ART initiation as appropriate. We assessed differences in proportions of partners completing each step in the HIV care cascade for partners of antenatal care and index clinic attendees.

### Cost analysis

The costing follows the approach taken by Mangenah and colleagues for costing HIVST [24] for estimating the cost of HIVST distribution. In brief, incremental economic costs were estimated from the provider’s perspective between April 2018 and March 2019. This comprised of capital cost items such as start-up training, building and storage, equipment and sensitization and recurrent cost items in the form of personnel, HIVST, other supplies, transportation, building operation and maintenance and other recurrent cost. Capital costs were annualized using a 3% discount rate, over their useful life span. All costs were estimated in 2018/2019 South African Rand (ZAR) and converted to US dollars (USD) using the period average exchange rate of 14 ZAR = 1 USD. Confirmatory testing costs were excluded. We estimated the economic costs by using a detailed expenditure analysis, complemented by activity-based observations (time-and-motion analysis) to account for shared resource activities and micro-costing. Costs were stratified summarized by district (CoJ, CoT and DKK).

## Results

A total of 14,473 HIVST kits were distributed across the twelve clinics (Table 1). A majority of the kits were distributed in CoJ (60%), with 36% distributed in CoT and 4% in DKK. Of the kits distributed via the ANC and HIV index models, 71% (10,319/14,473) were delivered to ANC clients, and 29% (4,154/14,473) were delivered to Index clients, to deliver to their sexual partners. The average distribution output for the different facilities per district varied, with implementation taking place over 18 months (May 2018 – October 2019) in COJ; 9 months (February 2019 – October 2019) in CoT and 5 months (April – August 2018) in DKK (Table 1). In all three districts, partners of clinic attendees were reached with HIVST largely via the ANC modality compared to HIV index modality.

**Table 1:** Distribution facilities and output

	CoJ		CoT		DKK		
Total distributed	8,700		5,238		535		
	ANC	HIV + Index	ANC	HIV + Index	ANC	HIV + Index	Total output by Facility
Bellavista clinic	185	17	-	-	-	-	202
Hillbrow clinic	1,831	425	-	-	-	-	2,256
Joubert Park clinic	1,249	1,954	-	-	-	-	3,203
Rosettenville clinic	177	36	-	-	-	-	213
Yeoville clinic	1,962	864	-	-	-	-	2,826
K T Motubatse clinic	-	-	1,134	358	-	-	1,492
Kgabo clinic	-	-	1,192	143	-	-	1,335
Laudium clinic	-	-	1,238	168	-	-	1,406
Olivenhoutbosch clinic	-	-	884	121	-	-	1,005
Grace Mokgomo clinic	-	-	-	-	145	35	180
JB Marks clinic	-	-	-	-	101	5	106
Jouberton clinic	-	-	-	-	221	28	249
Total output by modality	**5,404**	**3,296**	**4,448**	**790**	**467**	**68**	

Key: *ANC* Antenatal Clinic attendee secondary distribution modality, *HIV* + Index, HIV Positive Index partner modality

### Population reached

Of the 10,319 kits distributed via ANC clinic attendees, 10,256 (99.4%) went to male sexual partners of ANC clients, and 60 (0.6%) to female sexual partners. One HIVST recipient was transgender and 2 HIVST recipients’ gender were undisclosed, (Table 2). Most of the partners of ANC clients receiving HIVST kits were between 25 and 34 years (58.2%), with 1,281 kits (12.6%) distributed to people below 25 years, and 282 kits (2.8%) going to partners of ANC clients older than 45 years. Of the 4,154 HIVST kits issued to HIV index clients for use by their partners, 3,269 (78.7%) went to males, 880 (21.2%) to females, and five (1%) to transgender or to partners with no disclosed gender (Table 2).

**Table 2:** Demographic characteristics of recipients of partner provided HIVST kits

Distribution modality	**ANC**			**HIV Index**		
District	COJ	COT	DKK	COJ	COT	DKK
Setting	Urban	Sub-Urban	Peri-Urban	Urban	Sub-Urban	Peri-Urban
Total distributed	**5,404 (52.4%)**	**4,448 (43.1%)**	**467 (4.5%)**	**3,296 (79.3%)**	**790 (19.0%)**	**68 (1.6%)**
**Gender**						
male	5,368 (99.3%)	4,422 (99.4%)	466 (99.8%)	2,615 (79.3%)	596 (75.4%)	58 (85.3%)
female	36 (0.7%)	23 (0.5%)	1 (0.2%)	677 (20.5%)	193 (24.4%)	10 (14.7%)
transgender	0 (0.0%)	1 (0.0%)	0 (0.0%)	2 (0.1%)	1 (0.1%)	0 (0.0%)
undisclosed	0 (0.0%)	2 (0.0%)	0 (0.0%)	2 (0.1%)	0 (0.0%)	0 (0.0%)
**Age**						
< 20	43 (0.8%)	53 (1.2%)	19 (4.1%)	66 (2.0%)	11 (1.4%)	6 (8.8%)
20–24	542 (10.1%)	531 (12.0%)	90 (19.3%)	130 (3.9%)	59 (7.5%)	13 (19.1%)
25–34	3,170 (59.0%)	2,528 (57.2%)	214 (45.9%)	1,164 (35.3%)	316 (40.0%)	15 (22.1%)
35–44	1,442 (26.9%)	1,136 (25.7%)	116 (24.9%)	1,370 (41.6%)	272 (34.4%)	23 (33.8%)
> 45	141 (2.6%)	124 (2.8%)	15 (3.2%)	565 (17.1%)	119 (15.1%)	11 (16.2%)
Unknown/missing	30 (0.6%)	50 (1.1%)	12 (2.6%)	1 (0.0%)	13 (1.6%)	0 (0.0%)
Median Age ( IQR)	31 (8.0)	30 (8.0)	30 (10.0)	36 (10.0)	35 (11.0)	34.5 (17.0)

Under the HIV Index modality, 285 HIVST kits (6.9%) were issued for clients’ partners below 25 years, and 695 (16.8%) went to partners older than 45 years. Most kits went to partners between the ages of 25 and 34 years (36.1%) and 35 and 44 years (40.2%). The median age of partners reached through index testing was 5 years older compared to partners reached via the ANC modality across all three districts (Table 2).

### Client follow-up

The highest client follow-up rate in the three districts was seen in CoJ for both distribution models with 2,461 (46.4%) of the consenting 5,270 ANC attendees who were provided partner kits being successfully followed up and 42.9% (1,398 / 3,257) of consenting HIV index clients successfully followed up (Table 3). Client follow-up rates were lowest amongst HIVST recipients in DKK facilities (17.6% and 19.2% in the ANC and Index models respectively). In CoJ and CoT, the first follow-up call was made at approximately two weeks post distribution compared to four weeks in DKK. The average time to complete all three follow-up calls was sixty-eight days in CoJ, seventy-nine days in CoT and forty-nine days in DKK. The second and third attempts at telephonic follow-up resulted in significant increments in number of clinic attendees successful reached for follow up in both CoJ and CoT approximately a 10% increment in successfully reached consenting clinic attendees (Table 3). Conversely both the second and third attempts to follow-up consenting individuals yielded no significant change (approximately 1%) in clinic attendees successfully reporting on outcomes post kit distribution in DKK. The variation in time for follow-up calls is attributable to several factors, foremost being not having real time data from facilities. Delays in data capturing in certain facilities were primarily the reason for the follow-up calls taking place outside of the prescribed window.

**Table 3:** Client follow-up, partner uptake, use and entry into care

Distribution modality	ANC			HIV Index		
District	CoJ	CoT	DKK	CoJ	CoT	DKK
Total distributed	**5,368 (52.3%)**	**4,422 (43.1%)**	**466 (4.5%)**	**3,296 (79.3%)**	**790 (19.0%)**	**68 (1.6%)**
**Client follow-up**						
Consented to follow-up	5,270 (98.2%)	4,276 (96.7%)	408 (87.6%)	3,257 (98.8%)	757 (95.8%)	52 (76.5%)
Successful interview 1	1,478 (28.0%)	940 (22.0%)	64 (15.7%)	666 (20.4%)	117 (15.5%)	9 (17.3%)
Successful interview 2	479 (9.1%)	373 (8.7%)	3 (0.7%)	246 (7.6%)	56 (7.4%)	0 (0.0%)
Successful interview 3	490 (9.3%)	381 (8.9%)	5 (1.2%)	486 (14.9%)	68 (9.0%)	1 (1.9%)
Days to 1^st^ interview	16 (16.0)	17 (13.0)	30 (75.0)	17 (17.0)	15 (77.0)	30 (224.0)
Days to 2^nd^ interview	33 (42.0)	39 (37.0)	7 (5.0)	29.0 (28.0)	41 (19.5)	10.0 (6.0)
Days to 3^rd^ interview	9 (33.0)	21.5 (60.0)	11 (4.0)	33 (51.0)	22 (54.0)	11 (2.0)
Total successfully followed up	2,461 (46.4%)	1,702 (39.6%)	72 (17.6%)	1,398 (42.9%)	241 (31.8%)	10 (19.2%)
**Partner uptake & use**						
Offered partner kit	2,098 (85.2%)	1,320 (77.6%)	57 (79.2%)	1,114 (79.9%)	192 (79.7%)	6 (60.0%)
Partner took kit	2,047 (97.6%)	1,311 (99.3%)	51 (89.5%)	1,065 (95.6%)	186 (96.9%)	6 (100.0%)
Partner used kit	1,995 (97.5%)	1,285 (98.0%)	48 (94.1%)	1,019 (95.7%)	181 (97.3%)	6 (100.0%)
**Entry into Care**						
HIVST Reactive result	105 (5.3%)	52 (4.1%)	2 (4.2%)	247 (24.2%)	48 (26.5%)	2 (33.3%)
Attended confirmatory testing	85 (81.0%)	40 (76.9%)	2 (100.0%)	218 (88.3%)	41 (85.4%)	2 (100.0%)
Confirmed HIV +	80 (94.1%)	34 (85.0)	2 (100.0%)	218 (100.0%)	40 (97.6%)	2 (100.0%)
Initiated on ART	79 (98.8%)	34 (100.0%)	1 (50.0%)	216 (99.1%)	40 (100.0%)	2 (100.0%)
Time to ART initiation Median (IQR)	0 (0.0)	0 (0.0)	15 (0.0)	0 (0.0)	0 (0.0)	10.5 (7.0)

### Partner self-testing, use and entry into care -ANC clients

Of the 10,135 (98.2%) ANC clients who consented to telephonic follow-up across the three districts, 4,235 (41.8%) were successfully reached by telephonic follow-up (Table 3). The proportion of ANC clients reporting that they offered HIVST to partner was 82.1% (3,475) with 98.1% (3,409) of the offered partners taking the HIVST kit and.

97.6% (3,328) reporting partner use of the delivered HIVST kit. Although there was a higher proportion of partners offered HIVST in CoJ (85.2%) compared to DKK (79.2%) and CoT (77.6%); significantly fewer partners took up the offer in DKK (89.5%) compared to > 97% in both CoT and CoT (Table 3). Of the partners who were reported to have used the self-test kit; ANC clients reported that 159 (4.8%) obtained a reactive HIVST result and 127 (79.9%) attended clinic for confirmatory testing. The yield did not vary much across the three districts. Of the 127 partners reported to have attended confirmatory testing, 116 (91.3%) received a confirmed HIV diagnosis and 114 (98.3%) were reported initiated on ART.

### Partner uptake, use and entry into care—HIV index clients

Of the 4,066 (97.9%) Index clients who consented to follow-up across the three districts, 1,649 (40.6%) were successfully followed up (Table 3). Of the 1,649 Index clients reached, 1,312 (79.6%) had offered a self-test kit to their partner with 95.8% (1257) of the partners accepting the HIVST kit and 95.9% (1206) reported to have used partner provided HIVST. Partner delivered self-test kits amongst Index clients yielded 297 HIVST reactive results (yield 24.6%) with 261 (87.9%) of the Index client’s partners reported to have attended clinic for confirmatory testing and 260 (99.6%) were reported as a confirmed HIV diagnosis. 258 (99.2%) who received a confirmed HIV diagnosis were initiated on ART. Across all three districts, reported partner attendance for confirmatory testing was higher in the HIV index model compared to the ANC model, with reported confirmatory testing attendances of 85.4%, 88.3% and 100% for CoT, CoJ and DKK respectively (Table 3).

#### Average cost

The average cost per HIVST kit distributed via ANC clients and Index varied across all districts (Table 4). In COJ, the average cost per kit distributed was US$13.33 to ANC clients compared to US$10.36 to people living with HIV. A similar variation was observed in.

**Table 4:** Average cost per HIVST kit distributed in three districts in South Africa

District	DKK				COJ				COT			
	**ANC**	**%**	**Index**	**%**	**ANC**	**%**	**Index**	**%**	**ANC**	**%**	**Index**	**%**
**Volume**	**566**		**80**		**3,463**		**2,896**		**623**		**854**	
**Capital costs**												
Start-up training	$0,27	4,08%	$1,90	11,16%	$0,06	0,43%	$0,05	0,53%	$0,25	1,63%	$0,05	0,33%
Building & storage	$0,00	0,06%	$0,03	0,15%	$0,00	0,00%	$0,00	0,00%	$0,00	0,02%	$0,00	0,01%
Sensitisation	$0,05	0,76%	$0,00	0,02%	$0,02	0,17%	$0,01	0,10%	$0,11	0,74%	$0,00	0,02%
Start-up other	$0,01	0,10%	$0,02	0,10%	$0,01	0,10%	$0,01	0,10%	$0,02	0,10%	$0,02	0,10%
Equipment	$0,06	0,95%	$0,25	1,44%	$0,07	0,56%	$0,05	0,49%	$0,17	1,13%	$0,09	0,59%
**Total capital costs**	**$0,39**		**$2,19**		**$0,17**		**$0,13**		**$0,55**		**$0,15**	
**Recurrent costs**												
Personnel	$2,76	41,71%	$6,17	36,23%	$9,97	74,80%	$7,00	67,63%	$10,44	68,85%	$10,58	72,71%
Test kits	$2,24	33,90%	$2,24	13,16%	$2,24	16,80%	$2,24	21,63%	$2,24	14,77%	$2,24	15,40%
Other Supplies	$0,62	9,39%	$4,67	27,45%	$0,42	3,13%	$0,56	5,45%	$1,33	8,78%	$0,99	6,83%
Transportation	$0,02	0,23%	$0,04	0,23%	$0,03	0,23%	$0,02	0,23%	$0,04	0,23%	$0,03	0,23%
Building operation & maintenance	$0,35	5,29%	$1,11	6,53%	$0,03	0,26%	$0,03	0,32%	$0,04	0,23%	$0,03	0,24%
Other recurrent	$0.23	3.52%	$0.60	3.52%	$0.47	3.52%	$0.36	3.52%	$0.53	3.52%	$0.51	3.52%
**Total recurrent costs**	**$6,22**		**$14,83**		**$13,16**		**$10,21**		**$14,62**		**$14,38**	
**Average cost per test kit distributed**	**$6,61**	**100%**	**$17,02**	**100%**	**$13,33**	**100%**	**$10,34**	**100%**	**$15,17**	**100%**	**$14,55**	**100%**

DKK with the average cost per kit distributed amounting to US$6.61 and US$17.02 for ANC and Index, respectively. In COT, however, the average cost per kit distributed was comparable at US$15.17 and US$14.55 for ANC and Index clients, respectively. The marginal difference in the average cost per kit distributed in COT is attributed to a relatively similar total cost and distribution volumes. Overall, the average cost per test kit distributed across the three districts ranged from US$7.90 in DKK to US$14.81 in COT. In COJ, the overall cost per kit distributed was US$11.98.

## Discussion

To our knowledge, this is the first real-world implementation to target both ANC clients and people living with HIV for secondary distribution of HIVST to their sexual partners at this scale in South Africa. Though a larger proportion of ANC clients was reached compared to people living with HIV, the high number of people living with HIV who took the HIVST kit for their male partners demonstrates the acceptability and feasibility of implementing this strategy in routine care services. Distribution of HIVST kits to people living with HIV instead of only ANC clients to deliver to their partners has the potential to identify people living with HIV who are unaware of their HIV status. The primary measure of success for secondary distribution of HIVST for men via ANC and HIV index modalities from the STAR project perspective was to use HIVST kits to facilitate closing the testing gap by reaching high risk sexually active men, 20 years and older, as well as exposed sexual partners of index clients. Similar to findings from STAR projects targeting men in three other African countries [13], both ANC and HIV index HIVST distribution modalities reached men successfully (> 99% of kits distributed via ANC clinic attendees and > 75% of kits distributed via HIV index clients went to men). Given the known poor clinic attendance behaviours of men; this implementation demonstrates practicality of innovative approaches of reaching men with HIV testing using partner-delivered secondary distribution of HIVST kits.

A second measure of success of the programme was HIVST uptake by sexual partners. Consistent with other HIVST secondary distribution studies for male partners, there was a high uptake after offer and reported use of HIVST delivered by partners of ANC clients [25, 26]. The success of the program may be attributed the step-by-step explanation on performance of the test, in addition to support materials, video links, frequently asked questions, and instructions in their native language that primary recipients received upon receipt of the secondary test kit for partner distribution. However, at the time this study was conducted only one other study had targeted male partners of HIV index clients and no difference of adverse events was found between women who delivered HIVST kits and those who delivered standard partner referral slips to their partners [18]. Overall, there have been high uptake and low report of adverse events across HIVST secondary distribution studies, which may be a result of men’s preference for home-based testing, including HIVST, compared to standard facility-based testing [27, 28]. One study found that one of the reasons men prefer women-delivered HIVST kits is that this approach fit into their lifestyles which were characterized by extreme day-to-day economic pressures, including the need to raise money for food for their household daily [27]. The high uptake and use of HIVST kits, especially among partners of HIV index clients in our study and Dovel et al. [18], is promising and suggest the need for routine implementation approach and adaptation of such models in other settings.

A third measure of success of the programme was clinic attendance for confirmatory testing and ART initiation of partners who screen positive for HIV using the HIVST. ANC and Index clients reported a high proportion of their newly HIV diagnosed partners (> 85%) entering into care early after HIVST were delivered to their partners across both models. Although these findings are encouraging, they are based on self-report and future studies should attempt to measure these outcomes objectively. Among the few studies that have reached men who receive HIVST through their partners, uptake of confirmatory blood-based testing is much lower at 20% among male partners of HIV index clients [18]. Uptake of follow-up services was lower (18%) in another study that objectively measured linkage to care among men who received HIVST kits from their partners [26]. However, men who received HIVST kits from their partners and information about a financial incentive for visiting the clinic for follow-up services were more likely to attend the clinic for follow-up services [29], suggesting the need for additional interventions to facilitate linkage to care for male self-testers. Findings from a systematic review revealed that linkage to care among people newly diagnosed with HIV during home-based HIV counseling was often low when people were routinely referred to the clinic and higher when additional strategies were used to facilitate linkage [30]. Future efforts to reach male partners of ANC and HIV index clients should investigate different strategies that can facilitate and objectively measure clinic attendance for follow-up services.

The cost of facility-based HIVST distribution was highly variable between the three districts the average cost per kit distributed in CoJ and CoT is comparable to facility based HIVST distribution costs reported in other studies of similar settings [24]. The largest cost drivers for distributing the kits from a provider perspective was human resource across both distribution modalities and all three districts. The low distribution volumes in DKK also drove the higher average cost for HIVST distribution in DKK.

Lack of full knowledge on entry into care for positively screened partners is a limitation of this study and might have a strong impact on the generalizability of the reported metrices. In addition, we recognise low successful follow-up rates and that the findings are not generalizable as limitations of the study. However, we called all consenting clients multiple times in an effort to make the findings generally representative of the clients who agreed to be followed-up. Although consent to follow-up was sought at distribution and prior to conducting the telephonic survey, we acknowledge that the part of the innovation in HIVST is client convenience and confidentiality and our attempts to follow-up clients may have resulted in some clients not responding to the follow-up calls. Both obtaining and reporting sexual partner’s HIV test result opens to social desirability bias and non-response bias on the data we collected. We also observed higher follow up for CoJ facilities which can be attributed to the proximity of study staff to the clinics that allowed for more rigorous and frequent facility visits. Further, closer proximity to the clients by study staff linkage officers, ensured that follow up rates in this region were high and may not be replicable for all facilities that are not in close proximity for program implementers.

## Conclusions

Partner delivered self-testing was highly acceptable with high proportions of partners entering into care. HIV index testing delivered a high yield of HIV infected persons. Increasing successful follow-up rates can provide for more conclusive evidence on utilization of kits.

## Supplementary Information

**Additional file 1.**

**Additional file 2.**

## Data Availability

Any personally identifiable data cannot be made publicly available to protect participants’ privacy. All other relevant data are available upon request to the senior author (contact: mmajam@ezintsha.org).

## Abbreviations

ANC: Antenatal Care
ART: Antiretroviral Therapy
PLWH: People Living with HIV
HIVST: HIV Self-Testing
HTS: HIV testing services
WHO: World Health Organization
STAR: SA South Africa Self-Testing AfRica
CoJ: City of Johannesburg
CoT: City of Tshwane
DKK: Dr Kenneth Kaunda
ZAR: South African Rand
USD: United States Dollars
